# Modified ILM flap techniques versus classical inverted ILM flap technique for large macular holes: a systematic review and meta-analysis of randomized controlled trials

**DOI:** 10.1186/s40942-024-00567-z

**Published:** 2024-07-24

**Authors:** Sarah A. Alghamdi, Faisal F. Aljahdali, Rahaf K. Sharif, Jumanah J. Homsi, Asma A. Alzahrani, Lugean K. Alomari, Amro Abukhashabah

**Affiliations:** 1 Ophthalmology Residency Training Program in Western Region, Jeddah, Saudi Arabia; 2 College of Medicine, King Saud bin Abdulaziz University for Health Sciences, Jeddah, Saudi Arabia; 3 King Abdulaziz University, Jeddah, Saudi Arabia; 4 Medical Collage of Rabigh, King Abdulaziz University, Jeddah, Saudi Arabia; 5 Department of Ophthalmology, Ministry of the National Guard - Health Affairs, Riyadh, Saudi Arabia; 6 King Abdullah International Medical Research Center, Riyadh, Saudi Arabia

## Abstract

**Background:**

Macular holes (MHs) constitute a vitreoretinal interface disorder that occurs when structural abnormalities in the fovea lead to impaired central vision. The standard treatment for MHs is mainly surgical. Using an inverted internal limiting membrane (ILM) flap has enhanced the success rates of MH surgeries. This systematic review and meta-analysis aimed to compare the classical inverted ILM flap technique to modified ILM flap techniques for managing large MHs.

**Methods:**

We searched Medline, Embase, and CENTRAL. We included randomized controlled trials (RCTs) that compared the classic inverted ILM flap technique to modified ILM flap techniques as initial surgical treatment of eyes with large MHs of more than 400 microns. We sought to evaluate the following outcomes: (1) MH closure. (2) Best-corrected visual acuity (BCVA). (3) Foveal closure type (4) Rate of ellipsoid zone (EZ) defects and external limiting membrane (ELM) defects. The standardized mean difference (SMD) was used to represent continuous outcomes, while the risk ratio (RR) was used to represent dichotomous outcomes.

**Results:**

Four RCTs that enrolled 220 participants were deemed eligible. The analysis revealed no statistically significant differences in MH closure between both groups (95% CI: 0.20, 7.96; *P* = 0.81). No statistically significant differences in mean BCVA were found at 1 and 3 months between both groups (SMD: 0.04; 95% CI: −0.16, 0.23; *P* = 0.70 and SMD: −0.167; 95%CI: −1.240, 0.906; *P* = 0.760, respectively). In addition, there were no significant differences between the two groups in the pattern of foveal closure, namely U-shape, V-shape, and flap open at 3, 6, and 12 months (RR: 0.87; 95% CI: 0.67, 1.12; *P* = 0.28, RR: 0.96; 95% CI: 0.58, 1.61; *P* = 0.89, and RR: 1.95, 95% CI: 0.26, 14.50; *P* = 0.51, respectively). Finally, the analysis showed no statistically significant difference in both groups’ EZ and ELM defect rates at 3, 6, and 12 months (RR: 1; 95% CI: 0.85; 1.18: *P* = 1 and RR: 1.14; 95% CI: 0.90, 1.45; *P* = 0.27).

**Conclusion:**

Macular holes (MHs) constitute a vitreoretinal interface disorder that occurs when structural abnormalities in the fovea lead to impaired central vision. The standard treatment for MHs is mainly surgical. Using an inverted internal limiting membrane (ILM) flap has enhanced the success rates of MH surgeries. This systematic review and meta-analysis aimed to compare the classical inverted ILM flap technique to modified ILM flap techniques for managing large MHs.

## Introduction

A macular hole (MH) is a full-thickness loss of sensory retinal tissue that affects the anatomical fovea and primarily involves the foveola of the eye [1]. Frequently, MHs are unintentionally detected when a patient experiences blurry vision, with or without visual distortions, when covering the other eye. MHs can occur due to tissue absence, such as in traumatic MHs. Alternatively, they can result from retinal dehiscence, which is the case in idiopathic MH [2]. In a prospective study by Ezra, the prevalence of MH is estimated to be 33 per 100,000, and the percentage of bilateral MH is between 0% and 29% of patients. However, no MH occurred in the fellow eye if the vitreous was detached. They found the incidence was 15.6% in 5 years in contralateral eye [3].

Surgery is the conventional treatment approach for MH. The main principle underlying surgical MH management is the removal of all vitreoretinal tractional forces [4]. Pars plana vitrectomy combined with posterior vitreous detachment and internal limiting membrane (ILM) peeling are the primary procedures used to address various vitreoretinal disorders, including MHs. These procedures alleviate traction forces and help prevent the development of epiretinal membranes following surgery [5]. Moreover, the success rate of this procedure reaches up to 90%. However, this percentage decreases to 60% when the surgery is performed on patients with large MHs [4]. The inverted ILM flap technique was initially introduced by Michalewska et al. to treat large MHs measuring over 400 μm [6]. This innovative approach guarantees a closure rate of 98% for MHs and leads to notable functional improvements after the surgery [4]. However, it has been reported that patients are still at risk of having persistent MH after the surgery and may need another surgery to close the MH [7]. Other potential postoperative complications include elevated intraocular pressure, the formation of significant cataract, and the development of glaucoma [8].

The inverted flap (IF) technique is currently the preferred and most reliable approach for treating MHs. However, various modified technical variations of this procedure have been adopted in practice recently, including the temporal ILM flap technique, multi-layer ILM flap technique, inverted ILM flap without extra manipulation technique, and free flap technique, with varying post-surgery outcomes. Numerous clinical trials have been published comparing these techniques and their consequences for patients with large MHs. [9, 10] This systematic review and meta-analysis aimed to compare the classical inverted ILM flap technique with modified ILM flap techniques for managing large MHs and assess their anatomical and functional outcomes including MH closure, mean best corrected visual acuity (BCVA), foveal closure type, rate of ellipsoid zone (EZ) defects and external limiting membrane (ELM) defects.

## Methods

This systematic review and meta-analysis were carried out following a predetermined protocol registered with PROSPERO (CRD42023402058) and were conducted following the Preferred Reporting Items for Systematic Reviews and Meta-Analysis (PRISMA) guidelines.

### Eligibility criteria

**Patients** individuals with idiopathic large MHs > 400 microns; **Intervention**: modified ILM flap techniques; **Comparison**: classical inverted ILM flap technique; **Outcomes**: MH closure, mean best corrected visual acuity (BCVA), foveal closure type, and rate of ellipsoid zone (EZ) defects and external limiting membrane (ELM) defects. **Study design**: randomized clinical trials.

### Search strategy

The systematic search was conducted on Medline, Embase, and the Cochrane Central Register of Controlled Trials (CENTRAL) from their inception until February 15, 2023. No limitations were placed on publication dates, but only studies published in English were included. Medical Subject Headings (MeSH) terms were employed to optimize the search. The complete search strategy can be found in the supplementary material. In addition, ClinicalTrials.gov, the UMIN Clinical Trials Registry, the Australian New Zealand Clinical Trials Registry, the ISRCTN registry, and the MetaRegister of Controlled Trials were searched to identify ongoing or recently completed trials. The reference lists of the randomized controlled trials (RCTs) included in our study were also examined to identify any potentially relevant RCTs that may not have been captured in the systematic search.

### Study selection and data extraction

Randomly assigning pairs of reviewers: (S.A.G and R.K.S), (A.AZ. and L.K.A), and (J.J.H and F.F.A). Each two reviewers independently and in duplicate evaluated the titles and abstracts of eligible trials, examined the full text, and extracted relevant data. In instances of disagreement, a consensus was reached through discussion, or a third reviewer was consulted for resolution.

### Risk of bias assessment

The risk of bias in the included RCTs was evaluated based on the Cochrane Collaboration’s tool for assessing risk of bias. This tool comprises seven items: random sequence generation, allocation concealment (selection bias), blinding of participants and personnel (performance bias), blinding of outcome assessment (detection bias), incomplete outcome data (attrition bias), selective reporting (reporting bias), and other possible causes of bias [11].

### Meta-analysis

Weighted mean difference (WMD) or standardized mean difference (SMD) was used for analyzing continuous variables. Risk ratios (RRs) and their 95% confidence intervals (95% CIs) were used to report dichotomous outcomes. The mean change from the baseline was calculated based on the equations reported in the Cochrane Handbook for systematic reviews of interventions [12]. Data reported as median and range or mean and range were converted to mean and standard deviation (SD) based on Hozo et al.’s (2005) equation [13]. The fixed-effects model was used when a fixed population effect size was assumed. Conversely, the random-effects model was implemented when statistical heterogeneity was established. Statistical heterogeneity was calculated using Higgins’ I^2^ statistic and the Cochrane Q (chi-squared) test at values of > 50% and *P* < 0.10, respectively [14]. Subgroup analysis was conducted based on the type of modified flaps. Data analysis was performed using Review Manager version 5.4 (The Nordic Cochrane Centre, The Cochrane Collaboration, Copenhagen, Denmark) and Comprehensive Meta-Analysis v3 software [15, 16]. Significant differences were established at *P* < 0.05.

## Results

**Fig. 1:**
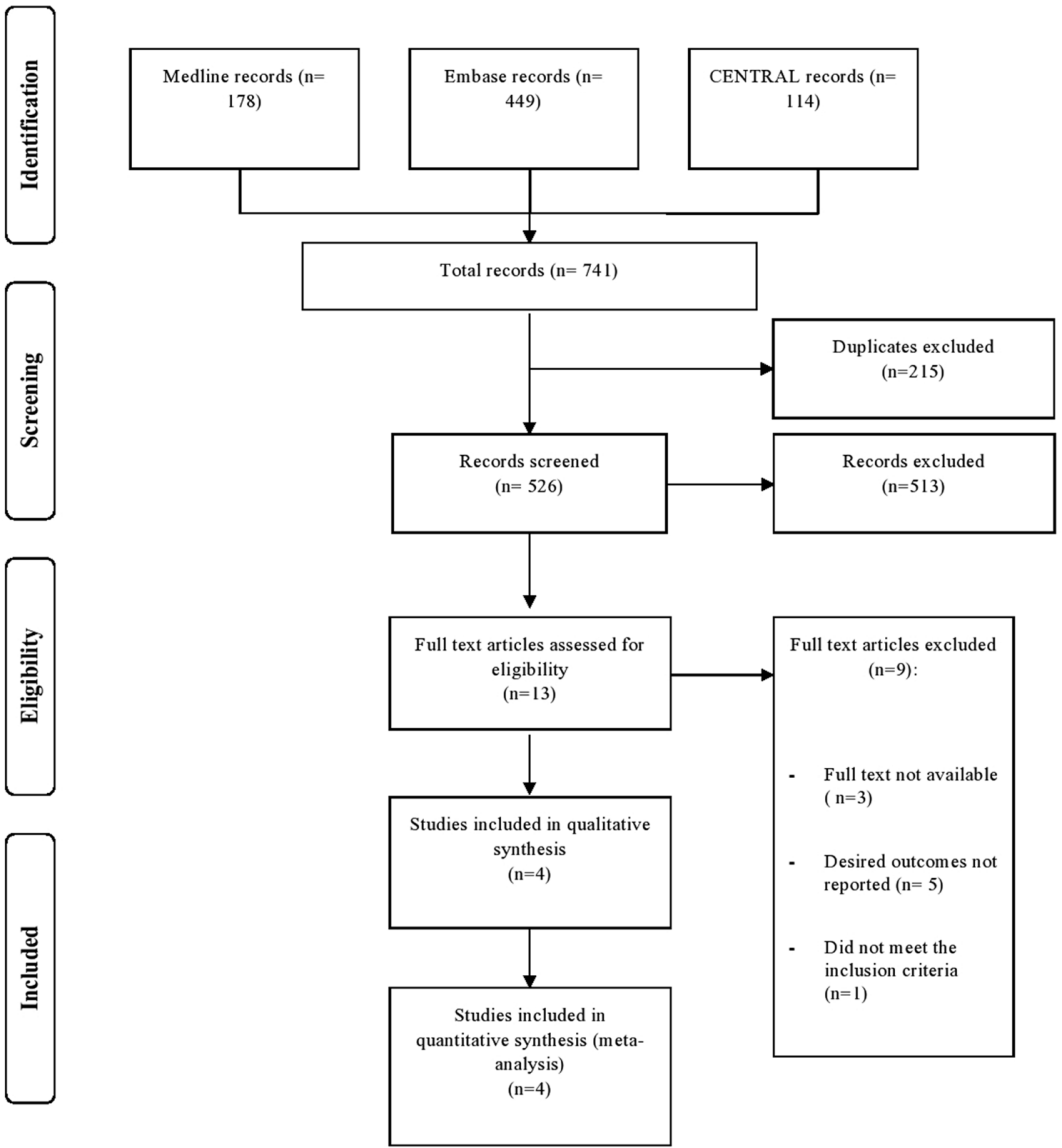
Shows the flowchart and study inclusion in this review

Figure 1 presents the PRISMA flowchart and the study selection process. The initial literature search identified 741 articles, of which 215 duplicates were removed. After screening the titles and abstracts, 513 articles were excluded, resulting in 13 articles for thorough evaluation of the full text. Finally, four articles representing four RCTs were considered eligible for inclusion [8, 17–19].

### Demographic characteristics of the studied groups

This meta-analysis included four articles, encompassing 220 patients with MH surgery [8, 17–19]. Of the included patients, 111 received modified flap techniques, whereas 109 received classic inverted ILM flap techniques for MH surgery. The average age of the patients ranged between 58.5 and 75 years in the modified flap group and from 64.2 to 73 years in the classic inverted ILM flap group. The mean MH width ranged from 544 to 884 and from 533 to 555 in the modified and classic groups, respectively. The pooled mean of MH width was 724.29 ± 295.20 in the modified flap group and 703.28 ± 240.04 in the classic inverted ILM flap group. The mean duration of full thickness MH ranged from 3.37 to 5.7 months in the modified flap group and from 3.06 to 6 months in the classic inverted ILM flap group. (Table 1).

**Table 1:**
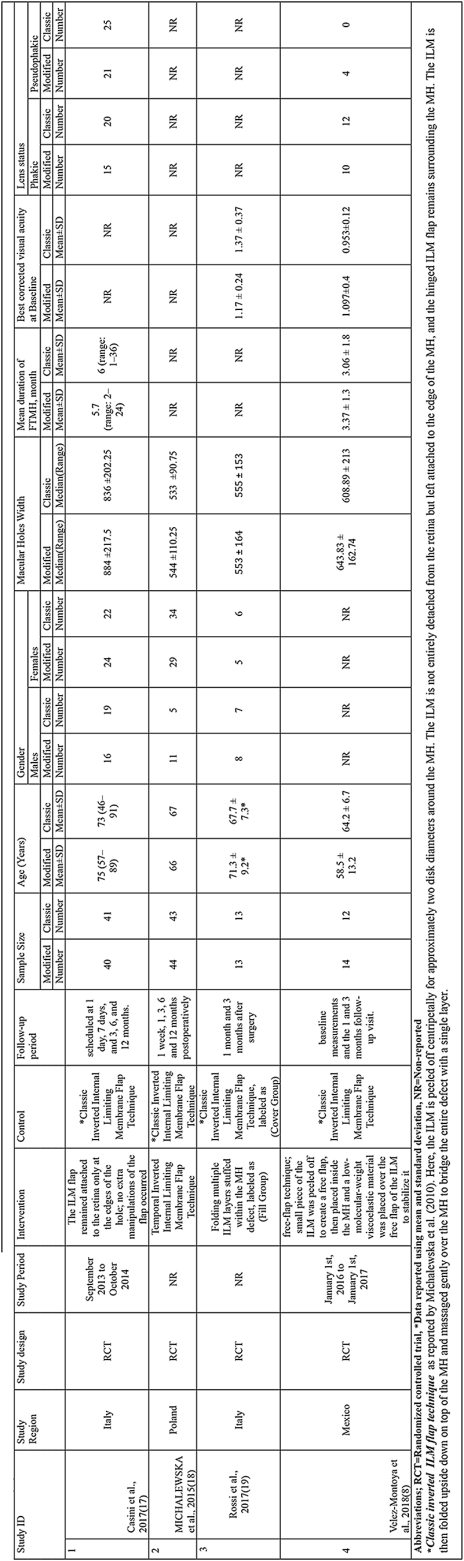
Demographic characteristics of the analyzed articles

### Risk of bias assessment

**Fig. 2:**
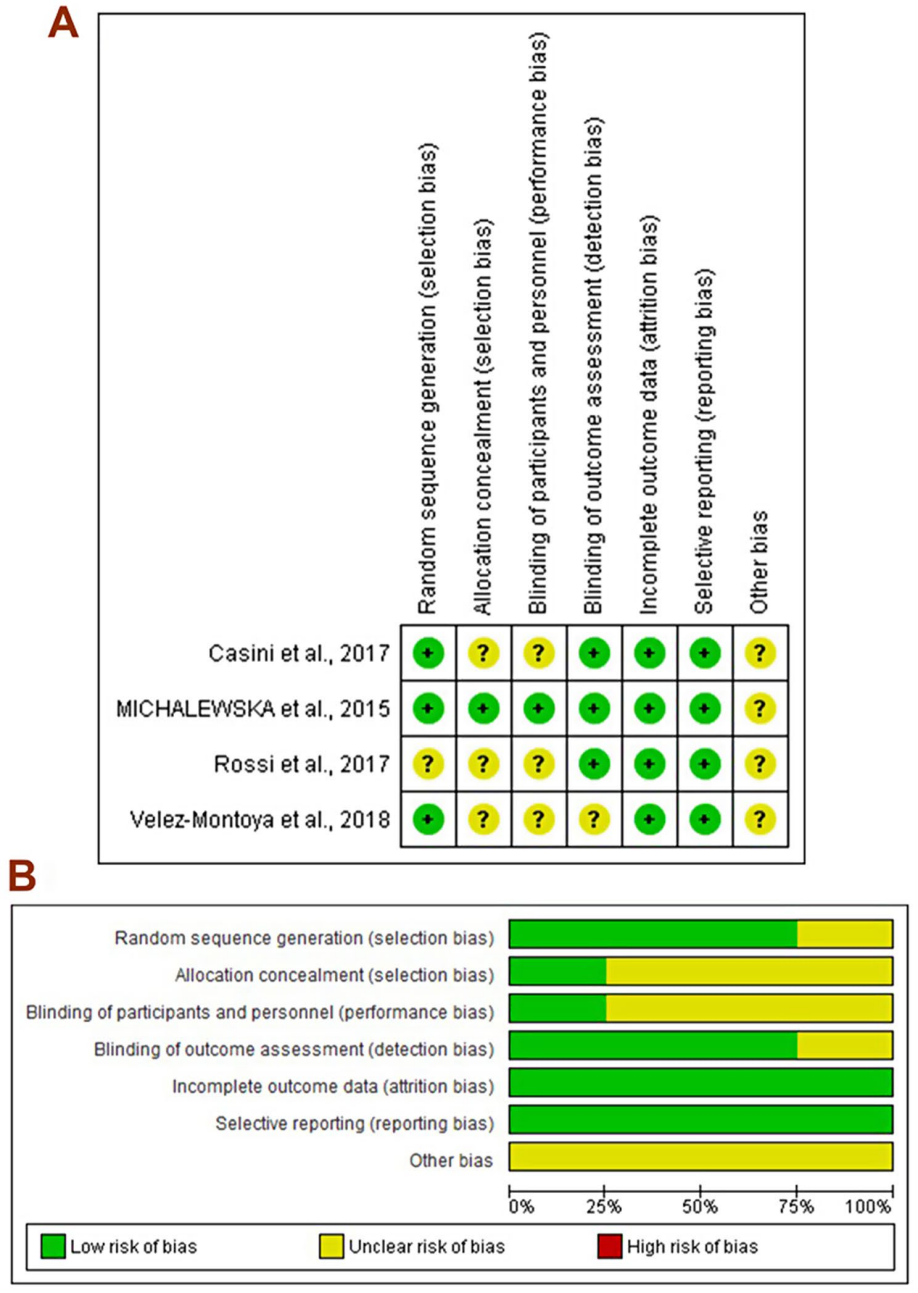
(**A**) Risk of bias graph (**B**) Risk of bias summary: review authors’ judgments about each risk of bias item presented as percentages across all included studies

All articles showed a low risk of random sequence generation bias [8, 17, 18] apart from that by Rossi et al. (2017). While three articles had an unclear risk of allocation concealment [8, 17, 19], one showed a low risk [18]. Three studies showed a low risk of detection bias [17–19]. All articles showed a low risk of attribution and reporting biases [17, 19]. None of the included studies reported their sample size calculation methods (Fig. 2).

### Study endpoints

#### MH closure

**Fig. 3:**
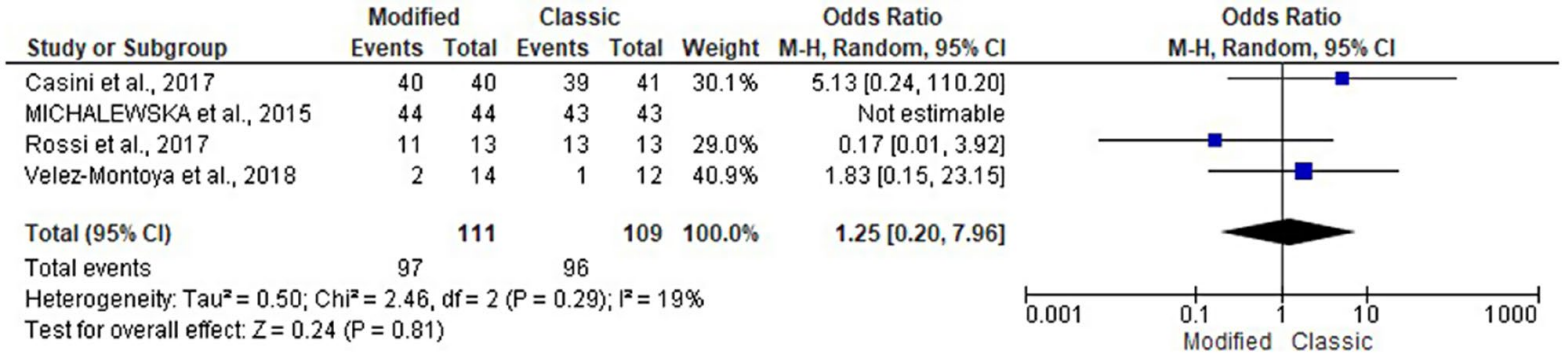
Forest plot of summary analysis of the odds ratio (OR) and 95% CI of the likelihood of macular hole closure between the modified and classic macular hole closure techniques. size of the blue squares is proportional to the statistical weight of each trial. The grey diamond represents the pooled point estimate. The positioning of both diamonds and squares (along with 95% CIs) beyond the vertical line (unit value) suggests a significant outcome (IV = inverse variance)

**Fig. 4:**
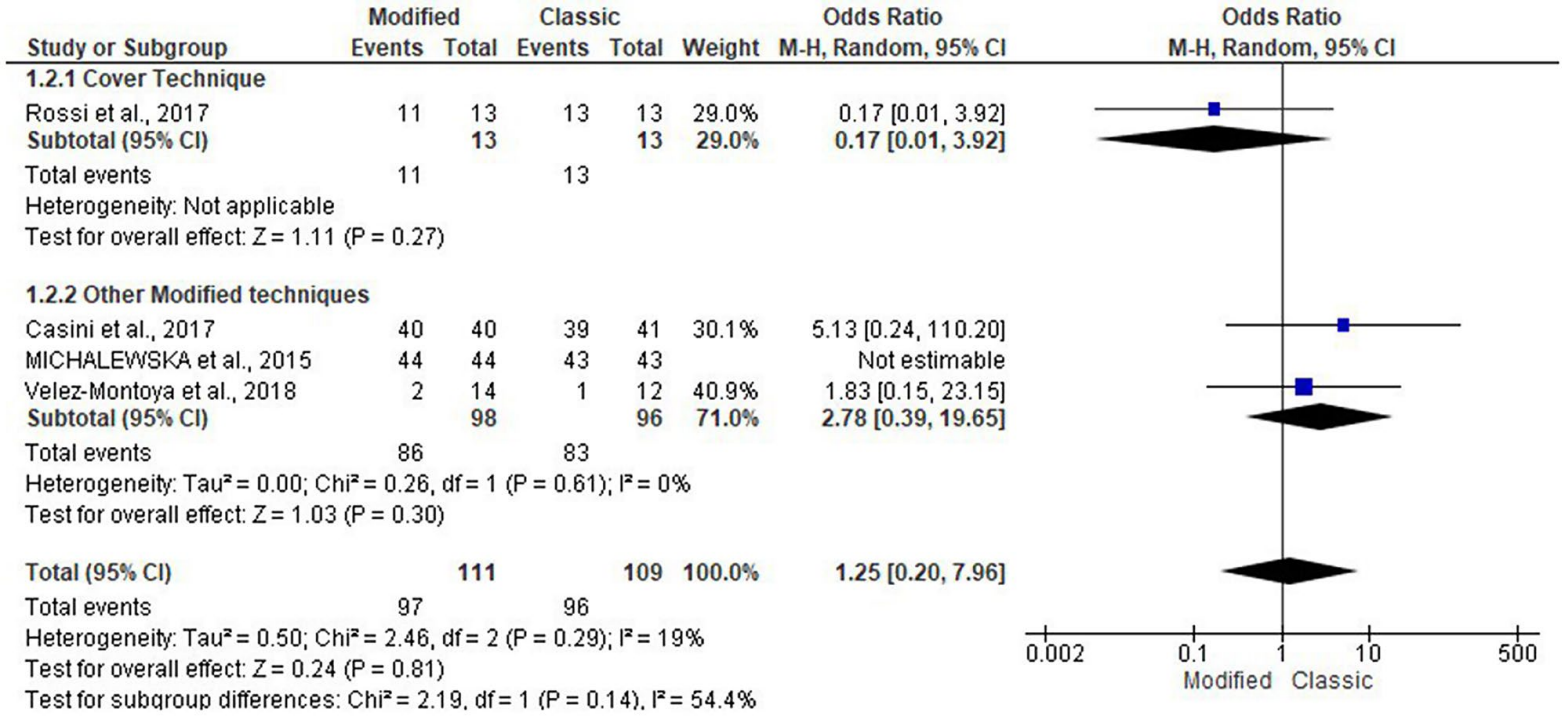
Forest plot of the subgroup analysis of the odds ratio (OR) and 95% CI of the likelihood of macular hole closure between the modified and classic macular hole closure techniques. size of the blue squares is proportional to the statistical weight of each trial. The grey diamond represents the pooled point estimate. The positioning of both diamonds and squares (along with 95% CIs) beyond the vertical line (unit value) suggests a significant outcome (IV = inverse variance)

The four articles included 220 patients, and the present study evaluated the likelihood of MH closure in the modified and classical groups [8, 17–19]. Pooling the data in the random-effects model (I^2^ = 19%; *P* = 0.29) revealed no statistically significant difference between both groups, with an odds ratio of 1.25 (95% CI: 0.20, 7.96; *P* = 0.81). Subgroup analysis based on the modified flap type revealed no statistically significant difference (Figs. 3 and 4**).**

### Mean change BCVA

#### At 1 month

**Fig. 5:**
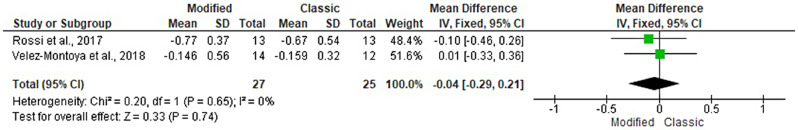
Forest plot of summary analysis of the Mean Difference (MD) and 95% CI of mean change from baseline of the best corrected visual acuity at one month between the modified and classic macular hole closure techniques. Size of the green squares is proportional to the statistical weight of each trial. The grey diamond represents the pooled point estimate. The positioning of both diamonds and squares (along with 95% CIs) beyond the vertical line (unit value) suggests a significant outcome (IV = inverse variance)

Two articles, including 52 patients, evaluated the mean change in the BCVA at 1 month [8, 19]. There was no statistically significant difference between modified and classical MH techniques (MD: −0.04; 95% CI: −0.29, 0.21; *P* = 0.74) with homogeneity between the analyzed articles (I^2^ = 0%; *P* = 0.65). See Fig. 5.

#### At 3 months

**Fig. 6:**
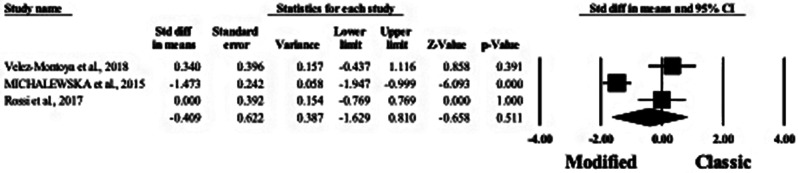
Forest plot of summary analysis of the Standardized Mean Difference (SMD) and 95% CI of the mean best corrected visual acuity at three month between the modified and classic macular hole closure techniques. size of the green squares is proportional to the statistical weight of each trial. the grey diamond represents the pooled point estimate. the positioning of both diamonds and squares (along with 95% CIs) beyond the vertical line (unit value) suggests a significant outcome (IV = inverse variance)

Three studies reported the BCVA difference at 3 months between modified and classical MH techniques [8, 18, 19]. In the random-effects model (I^2^ = 90.01%; *P* < 0.001), there was no statistically significant difference between both groups (MD: −0.167; 95% CI: −1.240, 0.906; *P* = 0.760). See Fig. 6.

### Patterns of foveal closure type

#### At 3, 6, and 12 months

**Fig. 7:**
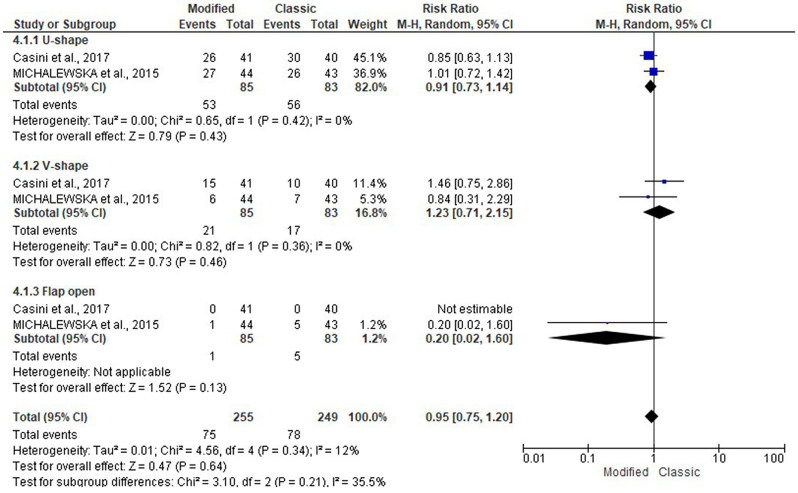
Forest plot of the subgroup analysis of the risk ratio (RR) and 95% CI of the pattern of foveal closure at three months between the modified and classic macular hole closure techniques. size of the blue squares is proportional to the statistical weight of each trial. the grey diamond represents the pooled point estimate. the positioning of both diamonds and squares (along with 95% CIs) beyond the vertical line (unit value) suggests a significant outcome (IV = inverse variance)

**Fig. 8:**
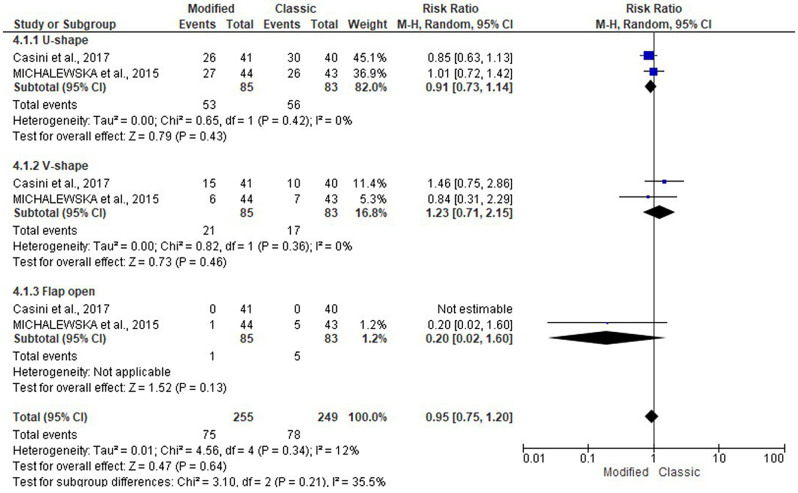
Forest plot of the subgroup analysis of the risk ratio (RR) and 95% CI of the pattern of foveal closure at six months between the modified and classic macular hole closure techniques. size of the blue squares is proportional to the statistical weight of each trial. the grey diamond represents the pooled point estimate. the positioning of both diamonds and squares (along with 95% CIs) beyond the vertical line (unit value) suggests a significant outcome (IV = inverse variance)

Two studies, including 168 patients, reported the pattern of foveal closure at 3, 6, and 12 months [17, 18]. At 3 and 6 months, subgroup analysis based on the pattern of closure revealed no statistically significant differences between modified and classical MH techniques regarding the risk of U-shape closure (RR: 0.91; 95% CI: 0.73, 1.14; *P* = 0.43), V-shape closure (RR: 1.23; 95% CI: 0.71, 2.15; *P* = 0.46), and flap open (RR: 0.20; 95% CI: 0.02, 1.60; *P* = 0.13). See Figs. 7 and 8.

**Fig. 9:**
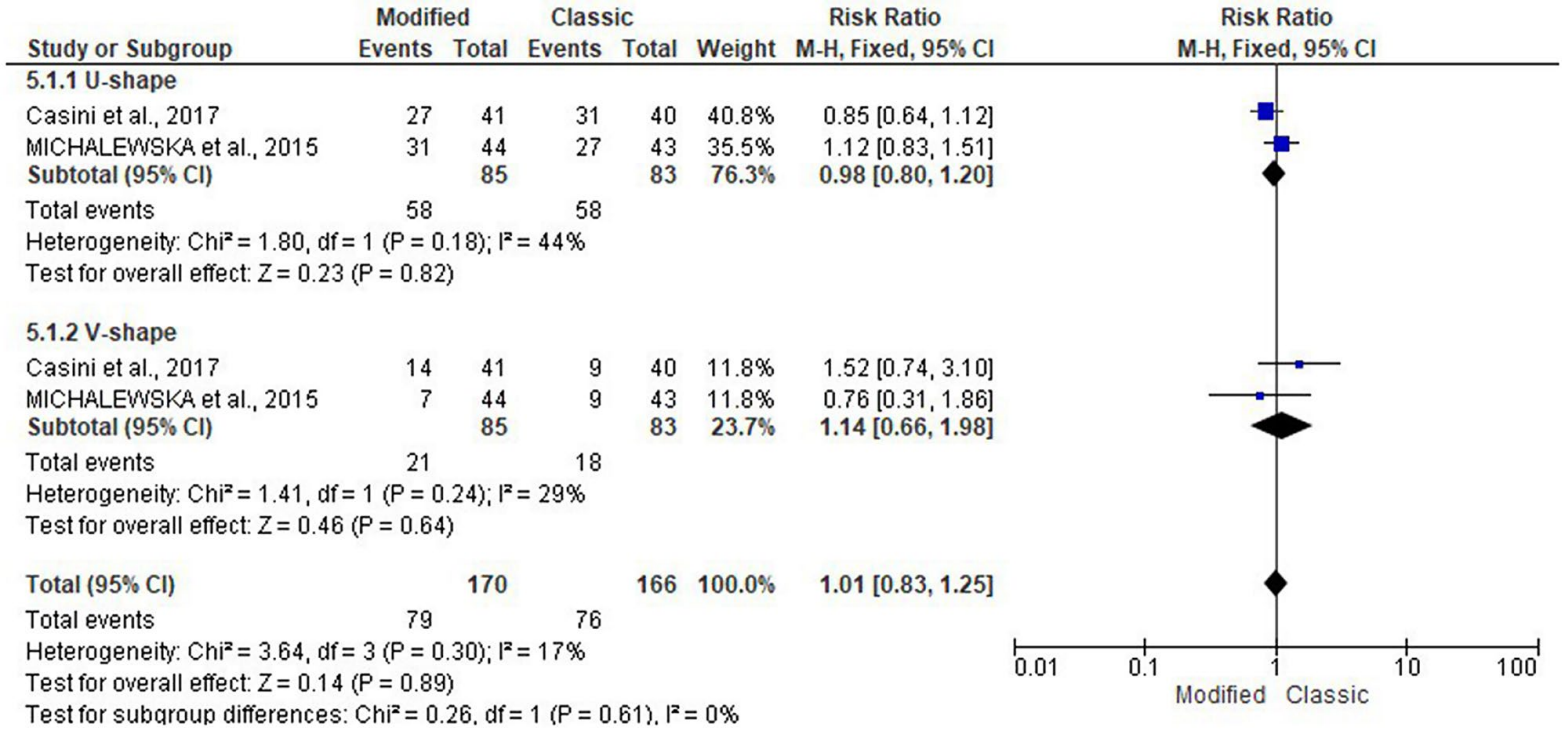
Forest plot of the subgroup analysis of the risk ratio (RR) and 95% CI of the pattern of foveal closure at twelve months between the modified and classic macular hole closure techniques. size of the blue squares is proportional to the statistical weight of each trial. the grey diamond represents the pooled point estimate. the positioning of both diamonds and squares (along with 95% CIs) beyond the vertical line (unit value) suggests a significant outcome (IV = inverse variance)

At 12 months, the risk of U-shape closure (RR: 0.98; 95% CI: 0.80, 1.20; *P* = 0.82) and V-shape closure (RR: 1.14; 95% CI: 0.66, 1.98; *P* = 0.64). See Fig. 9.

### Rate of EZ defects and ELM defects

**Fig. 10:**
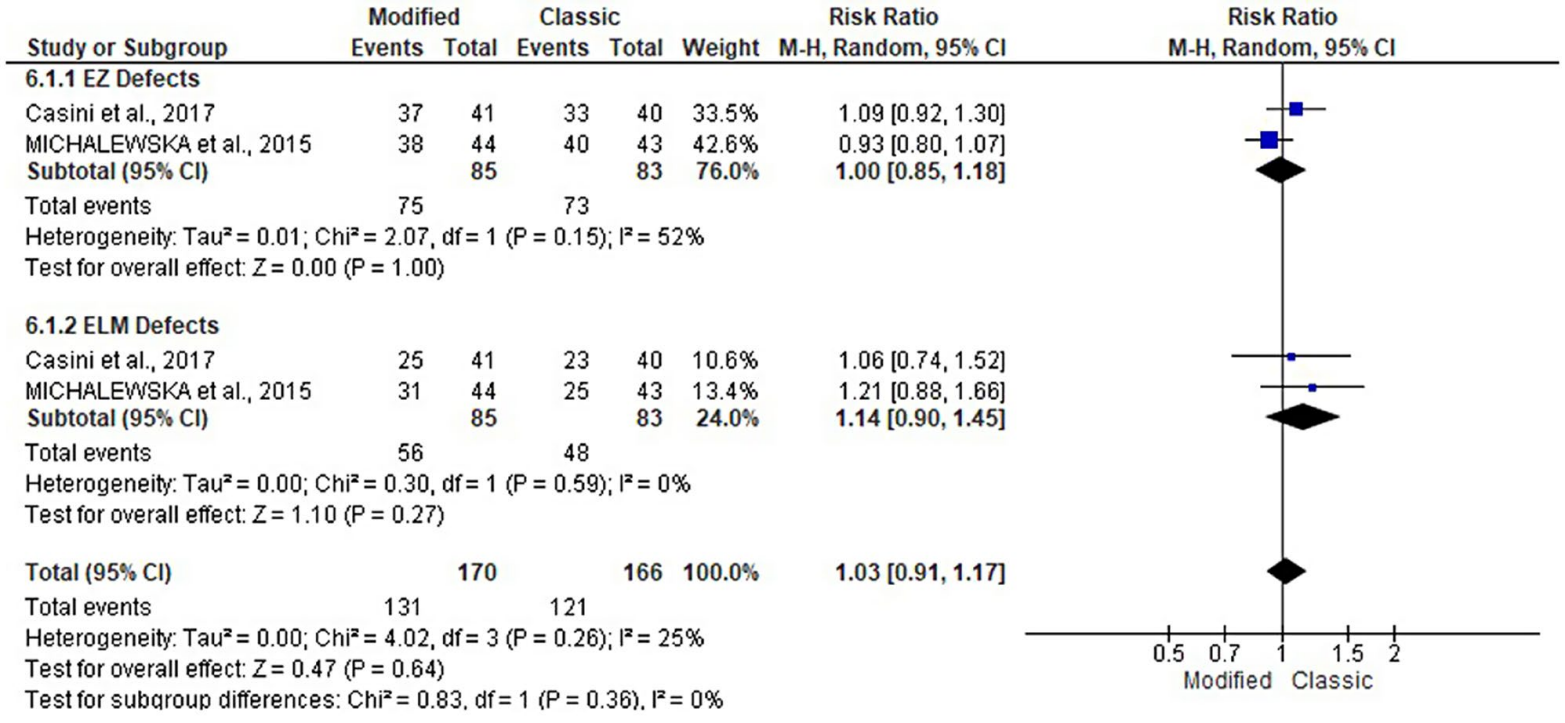
Forest plot of the subgroup analysis of the risk ratio (RR) and 95% CI of the rate of EZ and ELM defects between the modified and classic macular hole closure techniques at three months. size of the blue squares is proportional to the statistical weight of each trial. the grey diamond represents the pooled point estimate. the positioning of both diamonds and squares (along with 95% CIs) beyond the vertical line (unit value) suggests a significant outcome (IV = inverse variance)

**Fig. 11:**
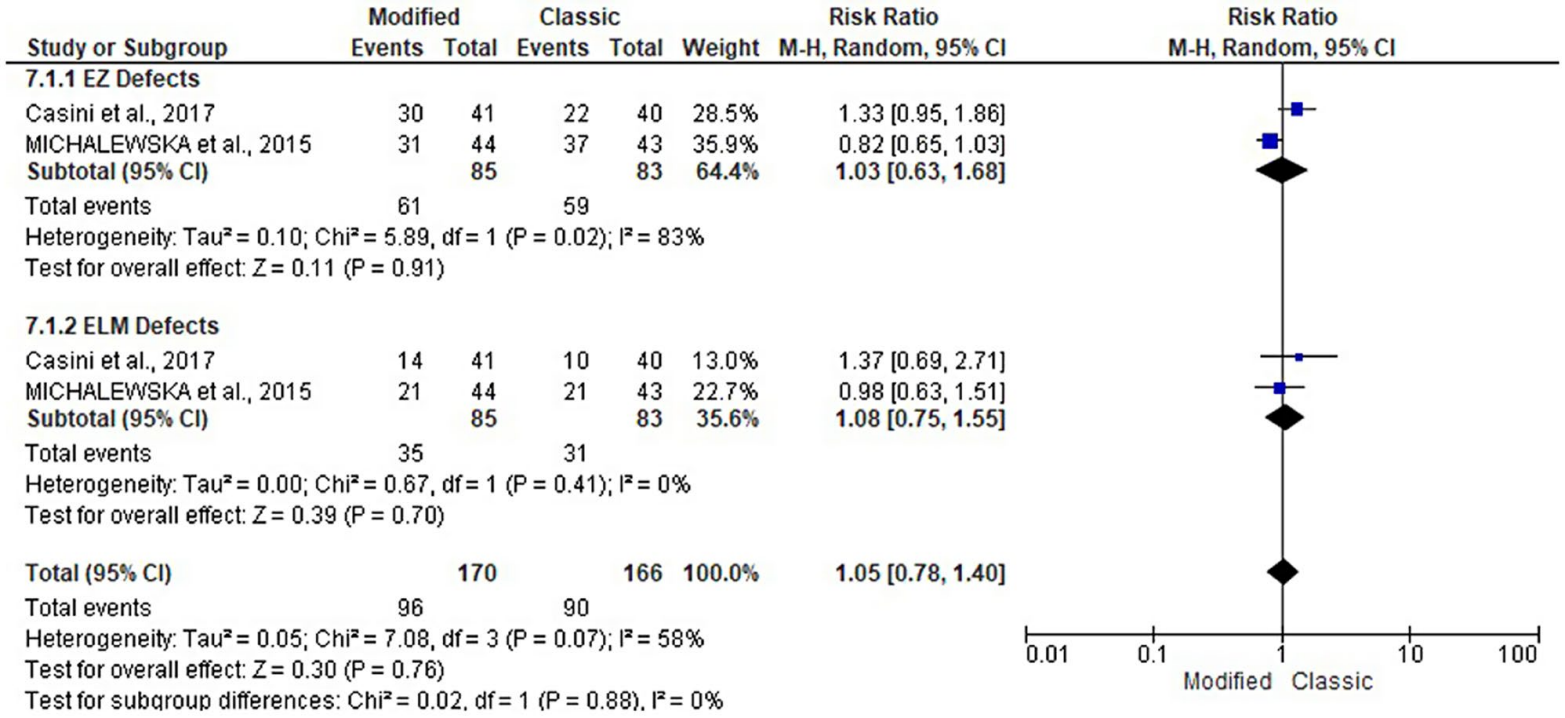
Forest plot of the subgroup analysis of the risk ratio (RR) and 95% CI of the rate of EZ and ELM defects between the modified and classic macular hole closure techniques at six months. size of the blue squares is proportional to the statistical weight of each trial. the grey diamond represents the pooled point estimate. the positioning of both diamonds and squares (along with 95% CIs) beyond the vertical line (unit value) suggests a significant outcome (IV = inverse variance)

**Fig. 12:**
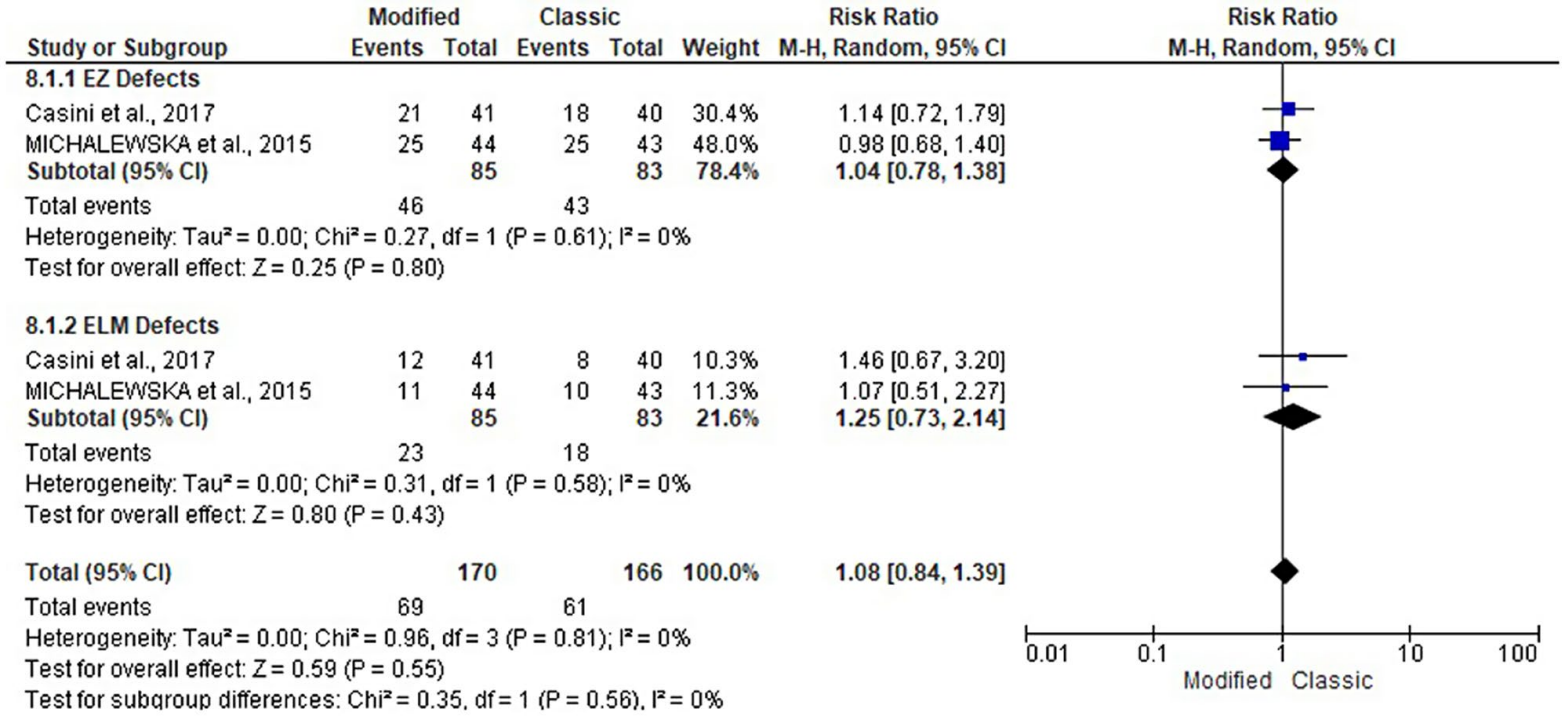
Forest plot of the subgroup analysis of the risk ratio (RR) and 95% CI of the rate of EZ and ELM defects between the modified and classic macular hole closure techniques at twelve months. size of the blue squares is proportional to the statistical weight of each trial. the grey diamond represents the pooled point estimate. the positioning of both diamonds and squares (along with 95% CIs) beyond the vertical line (unit value) suggests a significant outcome (IV = inverse variance)

The EZ and ELM defect rates were reported in two studies [17, 18], including 168 patients. They were evaluated at 3, 6, and 12 months following the modified and classical ILM flap techniques. Both groups had a similar pattern of EZ and ELM defects. At 3 months, the rate of EZ defects (RR: 1; 95% CI: 0.85, 1.18; *P* = 1) and ELM defects (RR: 1.14; 95% CI: 0.90, 1.45; *P* = 0.27). At 6 months, the rate of EZ defects (RR: 1.03; 95% CI: 0.63, 1.68; *P* = 0.91) and ELM defects (RR: 1.08; 95% CI: 0.75, 1.55; *P* = 0.70). At 12 months, the rate of EZ defects (RR: 1.04; 95% CI: 0.78, 1.38; *P* = 1) and ELM defects (RR: 1.25; 95% CI: 0.73, 2.14; *P* = 0.27). See Figs. 10, 11 and 12.

## Discussion

This review evaluated anatomical MH closure, BCVA, patterns of foveal closure types, and rate of EZ defects and ELM defects of large MH after initial surgery of the inverted ILM flap using either classical or modified techniques. The pooled MH closure rate revealed a similar closure rate using classical inverted ILM flap versus modified techniques. Three studies out of the four RCTs in the systematic review reported comparable results regarding anatomical closure rate between groups [8, 17, 18]. One study showed higher closure rates in the fill eyes group than the cover group (classical technique), although the differences were not statistically significant [19]. In general, the inverted flap technique was expected to have a high success rate of closure based on previous clinical trials, which have supported the role of the ILM flap technique in achieving better anatomical closure outcomes, especially for large MHs, compared to conventional ILM peeling alone [20–23]. As they believe that the ILM peeling released the tangential traction between epiretinal membrane (ERM) and ILM which is the main cause for the formation and expansion of MH [24]. They hypothesized that the ILM is a base membrane that provides both Muller cell fragments to stimulate glial cell proliferation and enhance MH closure and reestablish the foveal architecture [25]. Regarding BCVA, the mean values were not statistically significantly different between the study arms at 1 month and 3 months.

In the literature, we found two non-randomized comparative clinical trial. The first study conducted by Zgolli H, et al., they evaluated the four surgical techniques for large MH more than 400 microns; pars plana vitrectomy (PPV) with inserted flap ILM, PPV with classic inverted flap ILM peeling, PPV with inverted flap without manipulation of ILM flap (free flap technique) and PPV with temporal inverted flap ILM peeling. They found that the first group (inserted Flap) showed a poor restitution of the outer retinal layer which was correlated with compromised final BCVA despite closure of the MH. The other 3 techniques have comparable functional and anatomical results [4].

In Park JH, et al., the second non-randomized comparative trial. They included 41 eyes with minimum 500-micron MH, divided into two groups; ILM insertion technique and inverted ILM flap technique. Both techniques achieved a high closure rate and significant BCVA improvements. Although, consequently the postoperative BCVA and photoreceptor layer recovery were better in the inverted flap group, compared to the insertion group [26].

In aspect of the type of the tamponade and positioning after MH surgery, in Michalewska’s study, the eye was filled with air and in a prone position for 3–4 days, but in the work of Rossi and Casini, (sulfur hexafluoride) SF6 gas was used, with instructions to maintain a face-down position for 3–4 h a day for 3 days. In Montoya’s study, SF6 or (perfluoropropane) C3F8, according to the surgeon’s preference and face-down position for a minimum of 3 days was used.

There are several clinical trials and systematic review and meta-analysis studies support the face down position for large MH (≥ 400 μm), that showed significant MH closure rate compared to MH smaller than 400 μm with different duration of positioning using 3 days, 5 days, 7 days, 10 days, and 14 days of face-down positioning [27–31]. There is systematic review and meta-analysis study conducted by Dervenis, N. et al., they reviewed all types of tamponades that can be used in MH surgery including: SF6, C2F6, C3F8, air and silicon oil (SO) and their effect on MH closure and visual outcome. The differences were not statistically significant between any of the 3 comparison groups: SO vs. gases, SF6 vs. Air, and SF6 vs. C2F6/C3F8 regarding the MH closure rates or visual outcomes [32]. Another randomized prospective study compares the SF6 with C3F8 tamponade for small, medium, and large MH repair. Anatomical and visual outcomes were assessed. The outcomes in this study were similar in both types of tamponades independently of the stage of the MH [33].

An important tool to assess the outcome of the surgery is the anatomical closure of MH demonstrated by spectral domain OCT. Kang’s study categorized post-operative MH closure into two types depending on OCT: full MH closure without bare retinal pigment epithelium (RPE) and incomplete MH closure with bare RPE [34]. Masahito Imai published a more detailed classification based on the OCT appearance of MH closure: a U-type, which has a normal foveal contour, a V-type that has a steep foveal contour, and a W-type that indicates the presence of a foveal defect of the neurosensory retina. The former type was not documented in our reviewed RCTs as one of the foveal closure type outcomes [35]. Our meta-analysis contained two studies that assessed the type of MH closure on OCT test post-modified and classical MH surgery techniques. The analysis revealed no statistically significant differences between the two techniques regarding the type of foveal closure (U, V, or flap open) at 3, 6, and 12 months post-operation [17, 18]. This could be explained by the post-operative macular closure not being solely a consequence of the technique used during the surgery. Moreover, it depends on multiple factors such as chronicity and the size of the MH, as mentioned in Kang’s study [34]. Another essential anatomical structure that OCT can assess is the closure of the EZ and the ELM. Regarding visual outcome, previous studies found that a better visual result was associated with the restoration of these two structures; one of these studies by Taku Wakabayashi concluded that one of the crucial factors in predicting potential better visual acuity is the restoration of the ELM, which is critical for the re-functioning of the photoreceptors [36]. Yang found in his study that the EZ band’s thickness, integrity, and reflectivity were all strongly correlated with the postoperative BCVA outcome and patients with greater reflectivity in the EZ band and a smaller diameter of EZ disruption typically had better visual outcomes. Yang also mentioned that The EZ band is a promising indicator for the functional assessment of photoreceptors since it represents the recovery of their mitochondria [37].

Two of the included RCTs evaluated this outcome and found that the classical technique resulted in better EZ and ELM closure at 3, 6, and 12-months post-operation. However, the analysis revealed that the difference between the two techniques was statistically insignificant [17, 18].

And this is consistent with Ghassemi’s study who compared three different techniques of inverted ILM flap for the management of large idiopathic MH. His results revealed that There was no significant difference in likelihood of complete EZ regeneration and ELM recovery between the three groups. As well as the BCVA between the three modified techniques was statistically insignificant [38].

Different flap techniques had comparable results regarding closure rate, BCVA, type of foveal closure, and EZ and ELM restoration, indicating that the technique can be chosen based on the surgeon’s preference.

We acknowledge that our review has several limitations. First, incomplete data reporting in some studies could introduce outcome variability, affecting overall reliability. Second, the disparate follow-up durations limit the understanding of the long-term effectiveness of the surgical techniques. Third, the diversity in MH severity among the included studies must be uniformly addressed, impacting the comprehensive interpretation of our findings. Finally, the sample size was small as limited RCTs are published in this domain which can not be used to perform network meta-analysis.

Despite these limitations, our study provides valuable insights into MH surgeries, emphasizing the need for future research.

## Conclusions

This systematic review and meta-analysis provide valuable insights into the outcomes of inverted ILM flap techniques. The meticulous analysis of foveal closure patterns and the rates of EZ and ELM defects over 12 months demonstrates the consistency in outcomes between modified and classical MH surgery techniques. These comprehensive findings underline the reliability and efficacy of both approaches and emphasize the importance of tailored decision-making based on individual patient characteristics and surgical expertise to achieve optimal outcomes in MH surgeries.

## Data Availability

All data generated or analyzed during this study are included in this article and its supplementary material files. Further inquiries can be directed to the corresponding author.
